# The validation of the malay version of binge eating scale: a comparison with the structured clinical interview for the DSM-IV

**DOI:** 10.1186/2050-2974-1-28

**Published:** 2013-08-09

**Authors:** Sarah Anne Robert, Abdul Ghani Rohana, Zainudin Suehazlyn, Thambu Maniam, Shah Shamsul Azhar, Kamaruddin Nor Azmi

**Affiliations:** 1 Endocrine Unit, Department of Medicine, Universiti Kebangsaan Malaysia Medical Centre Jalan Yaacob Latif, Bandar Tun Razak, Cheras 56 000 Kuala Lumpur, Malaysia; 2 Department of Psychiatry, Universiti Kebangsaan Malaysia Medical Centre Jalan Yaacob Latif, Bandar Tun Razak, Cheras 56 000 Kuala Lumpur, Malaysia; 3 Department of Community Health, Universiti Kebangsaan Malaysia Medical Centre. Jalan Yaacob Latif, Bandar Tun Razak, Cheras 56 000 Kuala Lumpur, Malaysia

**Keywords:** Binge eating scale, Malay

## Abstract

**Background:**

The Binge Eating Scale (BES) questionnaire is a self-administered instrument developed to identify binge eaters. The aim of this study was to assess the validity of the Malay language version of BES as a screening instrument for binge eating. A cut-off point of 17 is taken as comparable to the Structured Clinical Interview for the DSM-IV patient version (SCID-I/P), the gold standard for the diagnosis of Binge Eating Disorder.

**Method:**

The questionnaire was structured from the English version of the original scale which has 16 items. The sample was obtained from outpatients and healthy adult volunteers at a teaching hospital. After completion of BES, the participants were interviewed with the SCID-I/P. The interviewer was blinded to the BES score.

**Results:**

The Malay version of BES yielded a sensitivity of 84.6%, specificity of 94.9%, a positive predictive value of 81.8%, a negative predictive value of 95.7%. Area under the curve was 0.95 (95% confidence interval: 0.90-0.99). The results of factor analysis indicated a two factor structure of feelings/cognition and behavioural manifestation of binge eating. Internal consistency, Cronbach’s alpha was 0.89.

**Conclusion:**

The BES performed satisfactorily as a valid instrument for screening of binge eating among Malay-speaking population.

## Background

Binge eating is currently increasing in recognition in association with higher body mass index (BMI), psychiatric symptoms, psychological disturbances, personality psychopathology and impaired quality of life [1,2]. It is a condition where a person recurrently has out-of-control eating episodes but without inappropriate compensatory behaviour. Binge eating disorder has been recognised as a diagnostic entity and included in the 4^th^ edition of Diagnostic and Statistical Manual of Mental Disorder (DSM-IV-TR)[3]. A binge episode is characterised by: (1) eating, in a discrete period of time (e.g. within any two hour period), an amount of food that is definitely larger than most people would eat during a similar period of time and under similar circumstances and (2) a sense of lack of control over eating during the episode (e.g. a feeling that one cannot stop eating or control what or how much one is eating) [3]. Binge eating was found in 7.5% to 30% of obese individuals seeking treatment [4-9] whist in community studies conducted in the United States, a lower prevalence was found, ranging from 1.0%-4.6% [9-11].

Structured clinical interview is considered the gold standard to assess any psychological disorder including binge eating. However, it is time-consuming, requires training and can only be administered to one subject at any particular time [12,13]. Therefore, self-administered questionnaires are useful alternatives used in studies on binge eating. The Binge Eating Scale (BES) is a 16-item self-administered questionnaire, designed specifically to identify the behavioural, emotional and cognitive characteristics of binge eating in individuals. Each item presents three or four differently weighted statements. Subjects would select the statement that best describes their perceptions and feelings about their eating behaviour. This questionnaire has been proven to be useful to identify binge eaters, to evaluate binge eating severity and also as a parameter of treatment outcome [14,15]. This measure has been found to discriminate effectively among individuals with absent, moderate, and severe binge eating problems. The authors of this measure reported that it yields internally consistent scores (Cronbach’s alpha = 0.85) [14]. Based on BES scores, the uncontrolled eating behaviour are graded into three different levels of severity: subjects scoring 17 and less were considered non-binge eaters, those scoring between 18 and 26 were moderate binge eaters and those scoring 27 and above were considered severe binge eaters [15,16]. Among the different validated versions in the literature are the English, Portuguese, Italian, and Spanish versions [6,12,14,17].

Due to the diversity of the Malaysian population and the use of the Malay language as the main language, we believed that it was necessary to have a validated Malay version of the BES to allow better understanding of the questions asked. The Malay version of the BES was obtained from a previous community survey study [18]. To our knowledge, there has been no validation study of the Malay version of BES using a gold standard interview such as the SCID-I/P. The aim of this study was to assess the validity of the Malay version of BES against the SCID-I/P.

## Method

### Participants

This was a cross sectional study carried out at a teaching hospital. We recruited 150 participants attending the medical outpatient clinic, medical students and staff members who were able to read and write in the Malay Language. Participants were excluded if they have serious medical illnesses that would affect their ability to answer the questionaire. Written informed consent was obtained from those who agreed to participate. Three ethnic groups were represented, the majority (76.7%) being Malay. The Malay Language ‘Bahasa Melayu’ is the national language for all ethnic groups to learn in school. It is the first language for the Malays. All our participants including the Chinese (17.3%) and Indians (6%) were fluent in the Malay Language.

### Procedure

Prior permission to use the questionnaire was obtained from the authors of the original English version of BES. Participants were initially asked to complete the Malay version of the BES. Participants were then interviewed with the Structured Clinical Interview for DSM-IV-patient version (SCID-I/P) by the investigator who was blinded to the BES scores. The investigator was trained by a senior psychiatrist familiar with the SCID-I/P. The BES questionnaire was then scored by an assistant who was blinded to the SCID-I/P results. The BES was scored by adding the values for the 16 individual items, with a final score varying from 0–46. The study protocol was approved by our Institutional Ethics and Clinical Research Committee (Approval number: FF-350-2012) and registered with the National Medical Research Registry (NMRR-12-825-13598). Participants received no compensation.

### Measures

As discussed above, the BES is a questionnaire that addresses behavioural manifestations (e.g. eating large amounts of food) and feelings/cognitions surrounding a binge episode (e.g. guilt, fear of being unable to stop eating). It consists of 16 items, eight describing the behavior manifestations and eight describing feelings/cognition. Each question had 3 or 4 weighted statements that reflect a range of severity for each characteristic. Weights were 0–3, (0 = indicates no binge eating problems, 3 = reflects severe binge eating problems). Participants choose the statement that best describes their perceptions and feelings about their eating behavior. For example, item number six has three statements that read: (1) I don’t feel any guilt or self-hate after I overeat (weight = 0); (2) After I overeat, occasionally I feel guilt or self-hate (weight = 1); and (3) Almost all the time I experience strong guilt or self-hate after I overeat (weight = 1).

The BES is scored by adding the individual values for the 16 items with the possible range of scores from 0–46. A cut-off point of 17 is commonly used; a score of 18 and above indicates the presence of binge eating.

The Structured Clinical Interview for the DSM-IV-patient version SCID I/P is a widely used clinician-administered interview for diagnosing psychiatric disorders according to DSM-IV Axis I.

### Statistical analysis

Data were analysed using IBM SPSS version 19. Concurrent validity was assessed in terms of sensitivity, specificity and predictive values. For these analyses, BES was compared to SCID-I/P, considered in this study the gold standard for diagnosis of BED. The Receiver Operator Characteristic curve (ROC curve) was used to describe the instrument’s performance. Construct validity was assessed using factor analysis. The internal consistency was evaluated by Cronbach’s alpha coefficient for the total score and score for each item.

## Results

### Participants

The study included 150 participants: 47 (31.3%) males, and 103 (68.7%) females, with age range from 18 years to 68 years (mean = 31 ± 0.898). There were 115 (76.7%) Malays, 20 (17.3%) Chinese and 9 (6%) Indians. A majority were from the middle income group (89.8%) and completed tertiary education (75.5%).

### Concurrent validity

**Figure 1:**
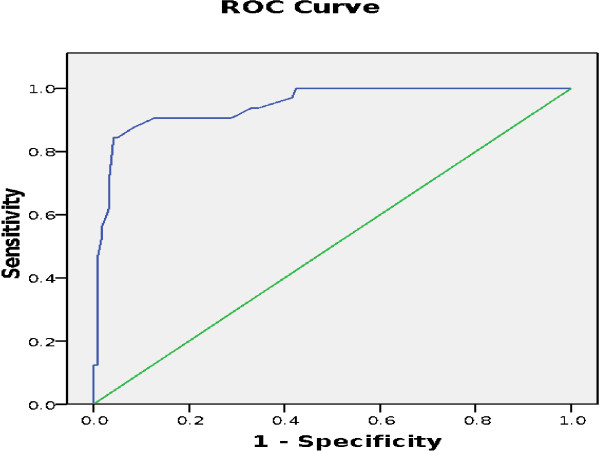
**ROC curve showing the accuracy of BES as a screening test for binge eating behaviour.** Area under curve = 0.95; 95% CI 0.90-0.99, S.E (area) = 0.021.

Table 1 shows the frequencies of the diagnosis of binge eating according to SCID-I/P and Malay version of the BES. The cut-off point of 17 is used; a score of 18 and above indicates the presence of binge eating. The summary of the concurrent validity results are presented in Table 2. The relationship between sensitivity and specificity is represented by the ROC curve. The area under the curve is 0.95 (95% CI: 0.90-0.99) (see Figure 1).

**Table 1:** BES score at a cut-off point of 17 and SCID-I/P diagnosis

		**SCID-I/P diagnosis**		
		**Binge eaters**	**Non-binge eaters**	**Total**
BES score	>17	27	6	33
	≤17	5	112	117
Total		32	118	150

SCID-I/P-Structured Clinical Interview for DSM-IV.

BES - Binge Eating Scale (Malay version).

**Table 2:** Prevalence of BED according to SCID-I/P and BES

	**%**	**95% Confidence intervals**
Sensitivity	84.6	68.2-93.1
Specificity	94.9	89.3-97.6
Positive predictive value	81.8	65.6-91.4
Negative predictive value	95.7	90.4-98.2
Prevalence	21	15.2-28.9
Accuracy	92.6	
Precision	81.8	

SCID-I/P Structured Clinical Interview for DSM-IV.

BES- Binge Eating Scale (Malay Version).

### Factor analysis

**Figure 2:**
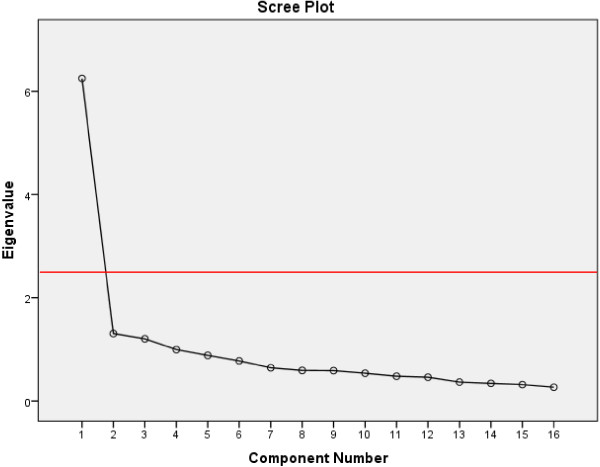
Scree Plot showing two components with eigenvalue more than 1.

Before performing factor analysis, the feasibility of parameter estimates and adequacy of measurement model were examined. Inspection of the correlation matrix revealed the presence of many coefficients of 0.3 and above. The Kaiser-Meyer-Olkin value was 0.89, more than the recommended value of 0.6 and Bartlett’s test of Sphericity was statistically significant, supporting the factorability of the correlation matrix. The two-factor structure was best fit to the data (see Figure 2). It explained a total of 47.2% of the variance, with factor 1 contributing 39.06% and factor 2 contributing 8.15%. Varimax rotation with two-factor solution was performed, revealing the presence of simple structure with both factors showing a number of strong loading as presented in Table 3. The factors were clearly feelings/cognitions related to binge eating (factor 1 items 1, 2, 6, 9, 11, 12, 14, and 16) and behavioural manifestations of binge eating (factor 2 items 3, 4, 5, 7, 8, 10, 13, and 15).

**Table 3:** Results of factor analyses: varimax rotated factor loadings

**BES items**	**Factors**	
	**1**	**2**
Item 12	**.778**	
Item 6	**.738**	
Item 1	**.670**	
Item 14	**.643**	
Item 2	**.604**	.330
Item 9	**.564**	.363
Item 11	**.559**	.311
Item 8		**.484**
Item 7		**.458**
Item 16	**.518**	.351
Item 3		**.684**
Item 15	.390	**.608**
Item 4		**.602**
Item 13		**.594**
Item 5	.370	**.545**
Item10	.429	**.465**

Note: Bolded items indicate major loadings for each item.

### Internal consistency

The overall internal consistency of the Malay version of the BES, measured by Cronbach’s alpha coefficient yielded 0.89. The corrected item-total correlation values for each item were more than 0.3 indicating that each item correlates well with the total score. The Cronbach’s Alpha for each item range from 0.874-0.891 (see Table 4). Mean of inter-item correlation was 0.337 with variance of 0.014.

**Table 4:** Results of reliability analysis, using Cronbach’s alpha

	**Item-total correlation**	**Cronbach’s alpha if item deleted**
Item 1	.446	.884
Item 2	.619	.877
Item 3	.534	.881
Item 4	.326	.887
Item 5	.563	.880
Item 6	.576	.879
Item 7	.633	.878
Item 8	.678	.874
Item 9	.601	.878
Item 10	.560	.880
Item 11	.555	.880
Item 12	.505	.882
Item 13	.291	.891
Item 14	.687	.875
Item 15	.619	.878
Item 16	.554	.880

## Discussion

Over the last decade, there has been a marked escalation of incidence of overweight and obesity worldwide. The World Health Organisation estimates that in 2008, 35% of adults and overweight and 11% are obese [19]. A national survey in 2006 has revealed a high prevalence of overweight and obesity of 44% among the adult population in Malaysia [20]. Binge eating is a recognised association with overweight and obesity, with a rate of 19% among healthy sedentary staff of a government institution [18] and 10.8% among patients attending a dietician clinic [21]. This emphasises the importance of identifying this particular eating habit among our population. Our choice of BES was based on the simple scoring system and its widespread use as a screening tool in various studies in the literature.

To the best of our knowledge, this current study is the first to compare the Malay version of BES with the standard SCID-I/P. The results showed that the Malay version of BES is a good tool to discriminate between binge eaters and non-binge eaters in our population with an AUC = 0.95 (95% CI: 0.90-0.99). It also showed high level of sensitivity in identifying individuals with binge eating behaviour (84.6%) and considerable specificity in identifying individuals without binge eating behaviour (94.9%). The findings of this study were fairly consistent with the validation study that used the Italian language version of BES, which was conducted among patients attending an outpatient clinic, yielding a sensitivity of 84.8%, and specificity of 74.6% [6]. However, a study that utilised the Portuguese language version [12] reported a higher sensitivity (97.8%) but lower specificity (47.7%) compared to ours and the Italian version. One reason for this variation could be the different recruitment strategies employed. The study that utilised the Portuguese version recruited participants through a newspaper advertisement to a eating disorder reference centre. Similarly, a higher sensitivity (94%) and lower specificity (76%) was also found in a recent study using the BES to screen patients seeking bariatric surgery in the United States of America [20].

Other studies that examines the validity of BES showed BES as a useful screening tool in identifying individuals with binge eating behaviour as well as being accurate in identifying individuals without binge eating behaviour. Two studies in the literature that compared BES with Eating Disorder Examination (EDE), another clinician directed interview showed that BES is an appropriate measure although with false positives being common. We did not use EDE in this study as it is too lengthy, taking as long as 60 minutes and we deemed this not practicable in an outpatient setting. The first study yielded sensitivity and specificity of 85% and 20% respectively [22] and second study yielded 93% and 49% [23]. Both studies had sensitivity comparable with ours but lower specificity which may be due to false positives. Screening instruments however, tend to tolerate a greater false positive rate. Accepting some false positives and then ruling out a true case in a complete interview is preferred rather than missing true cases completely as a result of a false negative screen.

The use of factor analysis helped to evaluate the factorial structure of the Malay version. Consistent with the literature, there were two main factors obtained which measures feelings/cognition and behavioural manifestations [24].

Internal consistency, a quantitative measurement using Cronbach alpha coefficient is to assess the consistency of results across the items within a test. The internal consistency reliability study of the Malay version of BES showed a value of Cronbach’s alpha of 0.89. Alpha coefficient ranges in value from 0 to 1 where the higher score indicates a more reliable scale. A value above 0.7 is indicated to be an acceptable reliability coefficient and thus our value of 0.89 fulfils this criterion [25]. This value is also consistent with the original BES which had a Cronbach’s alpha = 0.85 [14] and the Portuguese version, Cronbach’s alpha = 0.89 [12]. This demonstrates that the Malay version of the BES is a reliable screening tool for the Malay-speaking population.

This study has limitations. Although there was a good sample size, there was a low percentage of males and small representation from the lower socioeconomic group. This may limit the generalizability of these findings.

## Conclusion

This study demonstrated high levels of sensitivity (84.6%) and specificity (94.9%) of the Malay version of BES in detecting binge eating behaviour within a Malay-speaking adult population, with a positive predictive value of 81.8%, negative predictive value of 95.7% and Cronbach’s alpha of 0.89. In light of the increasing prevalence of overweight and obesity, binge eating may very well be an important factor. Therefore, the Malay version of the BES is useful as it has been demonstrated to be a valid and reliable instrument that is easy to administer.

## Competing interests

The authors declare that they have no competing interests.

## Authors’ contributions

SAR, TM, RAG, NAK, and SAS contributed to the study design, and data collection. SAR and SAS performed the statistical analyses, while RSA, TM, RAG, NAK, SZ participated in drafting and editing the manuscript. All authors read and approved the final manuscript.
